# Hypoxia-inducible factors: master regulators of hypoxic tumor immune escape

**DOI:** 10.1186/s13045-022-01292-6

**Published:** 2022-06-03

**Authors:** Qinghua Wu, Li You, Eugenie Nepovimova, Zbynek Heger, Wenda Wu, Kamil Kuca, Vojtech Adam

**Affiliations:** 1 College of Life Science, Yangtze University, Jingzhou, 434025 China; 2 MOE Joint International Research Laboratory of Animal Health and Food Safety, College of Veterinary Medicine, Nanjing Agricultural University, Nanjing, 210095 China; 3 Department of Chemistry, Faculty of Science, University of Hradec Kralove, 50003 Hradec Kralove, Czech Republic; 4 Department of Chemistry and Biochemistry, Mendel University in Brno, Brno, 613 00 Czech Republic; 5 Central European Institute of Technology, Brno University of Technology, Brno, 602 00 Czech Republic

**Keywords:** Hypoxia, Hypoxia-inducible factors, Tumor disease, Immunotherapy, Personalized medicine

## Abstract

Hypoxia, a common feature of the tumor microenvironment in various types of cancers, weakens cytotoxic T cell function and causes recruitment of regulatory T cells, thereby reducing tumoral immunogenicity. Studies have demonstrated that hypoxia and hypoxia-inducible factors (HIFs) 1 and 2 alpha (HIF1A and HIF2A) are involved in tumor immune escape. Under hypoxia, activation of HIF1A induces a series of signaling events, including through programmed death receptor-1/programmed death ligand-1. Moreover, hypoxia triggers shedding of complex class I chain-associated molecules through nitric oxide signaling impairment to disrupt immune surveillance by natural killer cells. The HIF-1-galactose-3-O-sulfotransferase 1-sulfatide axis enhances tumor immune escape via increased tumor cell-platelet binding. HIF2A upregulates stem cell factor expression to recruit tumor-infiltrating mast cells and increase levels of cytokines interleukin-10 and transforming growth factor-β, resulting in an immunosuppressive tumor microenvironment. Additionally, HIF1A upregulates expression of tumor-associated long noncoding RNAs and suppresses immune cell function, enabling tumor immune escape. Overall, elucidating the underlying mechanisms by which HIFs promote evasion of tumor immune surveillance will allow for targeting HIF in tumor treatment. This review discusses the current knowledge of how hypoxia and HIFs facilitate tumor immune escape, with evidence to date implicating HIF1A as a molecular target in such immune escape. This review provides further insight into the mechanism of tumor immune escape, and strategies for tumor immunotherapy are suggested.

## Background

In general, tumor occurrence is closely related to immune function [1, 2], as low or suppressed immune function increases the risk of tumor incidence [3]. Thus, scientists have proposed the “tumor immune editing” theory with regard to the relationship between tumorigenesis and immunity [4, 5]. According to this theory, the immune system can identify, monitor, and ultimately clear most malignant cells; however, a few malignant cells escape this surveillance and enter an “equilibrium” stage, during which the immune system and cancer cells modulate each other, resulting in no clinical symptoms [6, 7]. Malignant tumor cells have also evolved to bypass this equilibrium, culminating in immune escape [8, 9], which occurs through modifications to both the tumor cells and tumor microenvironment [10]. Knowledge of the mechanisms regulating tumor immune escape will contribute to the development of new strategies for immunotherapy [11]. As various tumor immune escape mechanisms exist in a complex network, comprehensive treatment targeting multiple escape mechanisms appears to be a promising strategy for drug development [12].

**Fig. 1:**
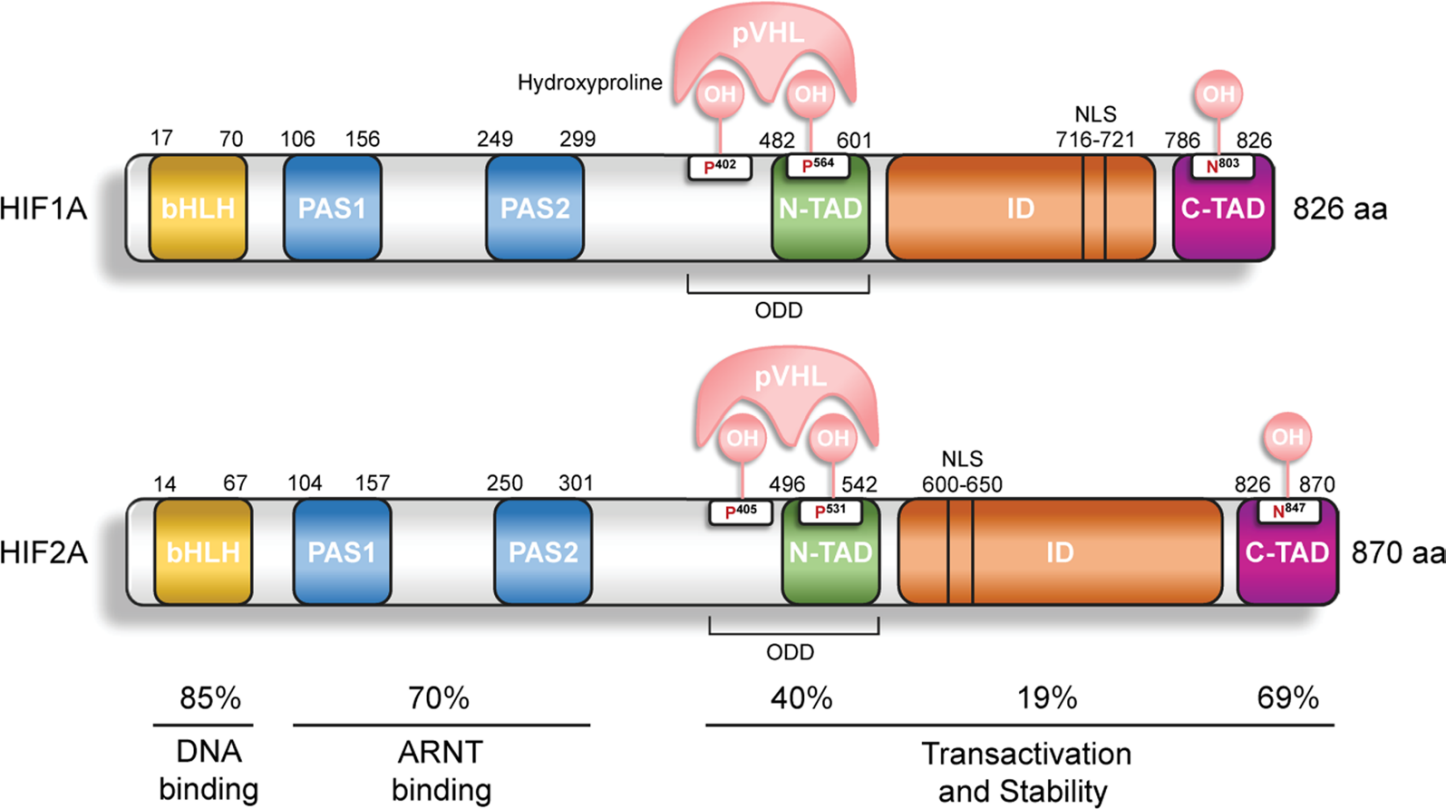
The protein structure of HIF1A and HIF2A. HIF1A and HIF2A contain basic helixloop-helix (*bHLH*) and Par-Arnt-SIM (*PAS*) transcription factors. And they also have N/C-terminal transactivation domain (*N*/*C-TAD*), inhibitory domain (*ID*), and oxygen-dependent degradation domain (*ODD*). Their DNA has a high degree of similarity. *NLS*: nuclear localization signal, *pVHL*: von Hippel-Lindau protein

Hypoxia is a common feature of the tumor microenvironment in various cancers [13, 14]. In most tumors, the degree of oxygenation is not uniform, and pathological hypoxic states can occur regionally [15]. Rapid tumor cells growth increases oxygen consumption during tumorigenesis, resulting in an intratumoral gradient of oxygen partial pressure [16]. Furthermore, hypoxia and overexpression of hypoxia-inducible factors (HIFs) 1 and 2 alpha (HIF1A and HIF2A) are involved in tumor immune escape and promote tumorigenesis [17–19] (Fig. 1). Under hypoxia, activation of HIFs and their downstream signaling pathways (including CXCR4, M-CSFR, and CD47) regulate the tumor-specific immune response, with production of several immunosuppressive cytokines and growth factors to allow for immune escape and promoted tumor progression [20, 21]. The immunological checkpoint composed of programmed cell death 1/programmed cell death 1 ligand 1 (PD-D1/PD-L1) inhibits T cell activation and proliferation, negatively regulating the cellular immune response and ultimately leading to immune escape [22, 23]. HIFs mediate tumor immune escape in various hypoxic solid tumors [24–26]. For example, HIF1A upregulates the negative immune checkpoint regulator V-set immunoregulatory receptor (VSIR) in colon cancer [27] and increases interleukin (IL)-23 expression in glutamine-deficient macrophages, which may suppress T cell immune function in clear cell renal cell carcinoma (ccRCC) to achieve immune escape [28]. In addition to HIF1A, HIF2A plays a role in the tumor immune escape mechanism: this isoform enhances expression of stem cell factor (SCF) in ccRCC patients, and upregulation of SCF protein expression promotes secretion of transforming growth factor (TGF)-β and IL-10, thereby forming an immunosuppressive tumor microenvironment and escaping tumor immunity [29].

Furthermore, tumor-associated long noncoding RNAs (lncRNAs) play pivotal roles in HIF1A pathway regulation and tumor immune escape. Under hypoxia, lncRNAs act as oncogenic HIF1A targets to promote tumor progression in osteosarcoma cells and bladder tumors [30, 31]. Notably, lncRNAs participate in tumor immune escape by influencing regulatory T cells (Tregs) and the PD-L1/PD-1 immune checkpoint to inhibit T cell immune functions [32]. Nevertheless, the underlying mechanism by which hypoxia-related lncRNAs promote immune escape is largely unknown. Indeed, most lncRNA studies have focused on their effects on the proliferation, invasion, and migration of tumors, whereas in-depth evaluation of their effects is required for targeting them clinically [33].

Tumor invasiveness and metastasis are major challenges in cancer treatment [34–36]. HIFs regulate a series of signaling pathways to promote the angiogenesis, metastasis, and invasive abilities of cancer cells [37–39]. Although tumor treatment strategies targeting HIFs have attracted widespread attention [40, 41], resistance is a common challenge, and hypoxia is a major factor that induces tumor chemoresistance [42]. By inducing expression of drug carrier proteins, the hypoxic tumor environment also affects drug transport and cellular drug uptake, further exacerbating resistance to chemotherapy [43].

This review discusses the underlying mechanisms by which hypoxia and HIFs (HIF1A and HIF2A) promote tumor immune escape, as well as the role of HIFs in cancer invasiveness, metastasis, and tumor chemoresistance. In addition, lncRNAs and their potential roles in hypoxic tumor immune escape are discussed. This review expands our understanding of immune escape in tumorigenesis and provides insight into potential novel and more efficient anticancer therapies.

## Tumor immune escape

Tumor immune escape is a phenomenon through which tumor cells escape recognition and attack by the immune system through various mechanisms, enabling their survival and proliferation [44, 45]. Through its immune surveillance function, the immune system recognizes malignant cells as “nonself” and precisely eliminates them, which prevents tumor development [46, 47]. Several immune escape mechanisms, including antigenic deletion, immune inhibition caused by tumor cells, tumor cell leakage, lack of costimulatory signals on the tumor cell surface, and the antiapoptotic effects of tumor cells, have been described thus far [48–50]. Tumor cells inhibit T cell activation through a series of changes (such as loss of antigen expression) to avoid immune surveillance and reduce the time elapsed before growth [51]. Tumor cells also regulate their autoantigenicity through antigen endocytosis or shedding [52]. Mutations in tumor-related genes alter signaling pathways in tumor cells, affecting the biological characteristics of the tumor and its immunogenicity [53, 54]. In general, a tumor antigen presented in the absence of a costimulatory signal will induce antigenic tolerance in the T cells that recognize the antigen [55]. Moreover, tumors produce immunosuppressive molecules, including indoleamine 2,3-dioxygenase 1 and PD-L1, which directly suppress the immune response or recruit immunosuppressive cytokine-secreting Tregs [56, 57]. Importantly, tumor cells also secrete various molecules, such as collagen, to generate a surrounding physical barrier [58] that prevents lymphocytes and antigen-presenting cells from infiltrating the tumor [59]. In the tumor microenvironment, immune cells, such as tumor-associated macrophages and T cells, are often suppressed by the actions of cytokines and nearby tumor cells, preventing tumor elimination and promoting tumor cell growth and metastasis [60, 61].

Signal transduction pathways in tumor cells promote release of various cytokines, chemokines, prostaglandins, and other inflammatory mediators into the tumor microenvironment. These inflammatory mediators bind to tumor cell surface receptors to activate intracellular signaling cascades, regulating gene expression to maintain tumor growth and invasion [62]. In addition, these mediators cause accumulation of oxygen and nitrogen free radicals, which increases microenvironmental oxidative stress and inhibits immune cell function [58, 63]. Activation of certain pathways, such as the signal transducer and activator of transcription 3 pathway, increases expression of inhibitory cytokines, such as TGF-β, IL-6, and vascular endothelial growth factor (VEGF), inhibits dendritic cell maturation, promotes Tregs aggregation, induces an immunosuppressive microenvironment, and suppresses the cytotoxic effects of natural killer (NK) cells and neutrophils, the effects of which promote immune escape [64–67].

Recent studies have shown that PD-1 and PD-L1 are closely related to tumorigenesis and tumor development [68]. PD-1 acts as a crucial inhibitory immune checkpoint molecule in the T cell-mediated immune response [69]. Tumor cells bind to PD-1 on tumor-infiltrating lymphocytes via PD-L1. This interaction induces lymphocyte apoptosis, allowing tumor cells to resist destruction and achieve immune escape [70, 71]. The PD-L1/PD-1 signaling axis mediates immune escape in the tumor microenvironment [68]; PD-L1 is selectively expressed on the surface of cancer cells, and its binding to PD-1 on the surface of activated T cells results in negative regulatory signal transmission, which decreases immune activity [72, 73]. However, targeted inhibition of PD-L1/PD-1 signaling reverses T lymphocyte suppression by tumor cells and enhances their recognition as well as the cytotoxicity of the immune system toward them [74]. Tumor cells also inhibit tumor-infiltrating T cells by releasing exosomes rich in surface PD-L1; these findings suggest that PD-L1 is present not only on the tumor cell membrane but also exists on tumor exosomal membranes in large amounts [75]. HIF1A positively regulates PD-L1 levels, indicating that HIF1A and hypoxia-induced upregulation of PD-L1 expression constitute a mechanism of tumor cell immune escape [24, 76]. Hypoxia causes rapid upregulation of PD-L1 expression in pulmonary pleomorphic carcinoma cells [24]. Furthermore, hypoxic tumor cells surrounding fusion necrosis coexpress HIF1A and PD-L1. In addition, patients with positive PD-L1 expression have a poor prognosis [24], and necrosis-surrounding PD-L1-positive tumor cells trigger PD-1-related T cell apoptosis and are resistant to immune-induced tumor death [24]. It has also been shown that *PD-L1* is a direct target of HIF1A [25]; HIF1A directly binds to the transcriptionally active hypoxia response element in the *PD-L1* proximal promoter, which activates its expression. Blocking PD-L1 under hypoxic conditions enhances MDSC-mediated T cell activation, accompanied by reduced expression of IL-6 and IL-10 in MDSCs [25]. Thus, inhibiting immune escape by blocking the HIF1A pathway is a promising strategy for anticancer therapy.

**Fig. 2:**
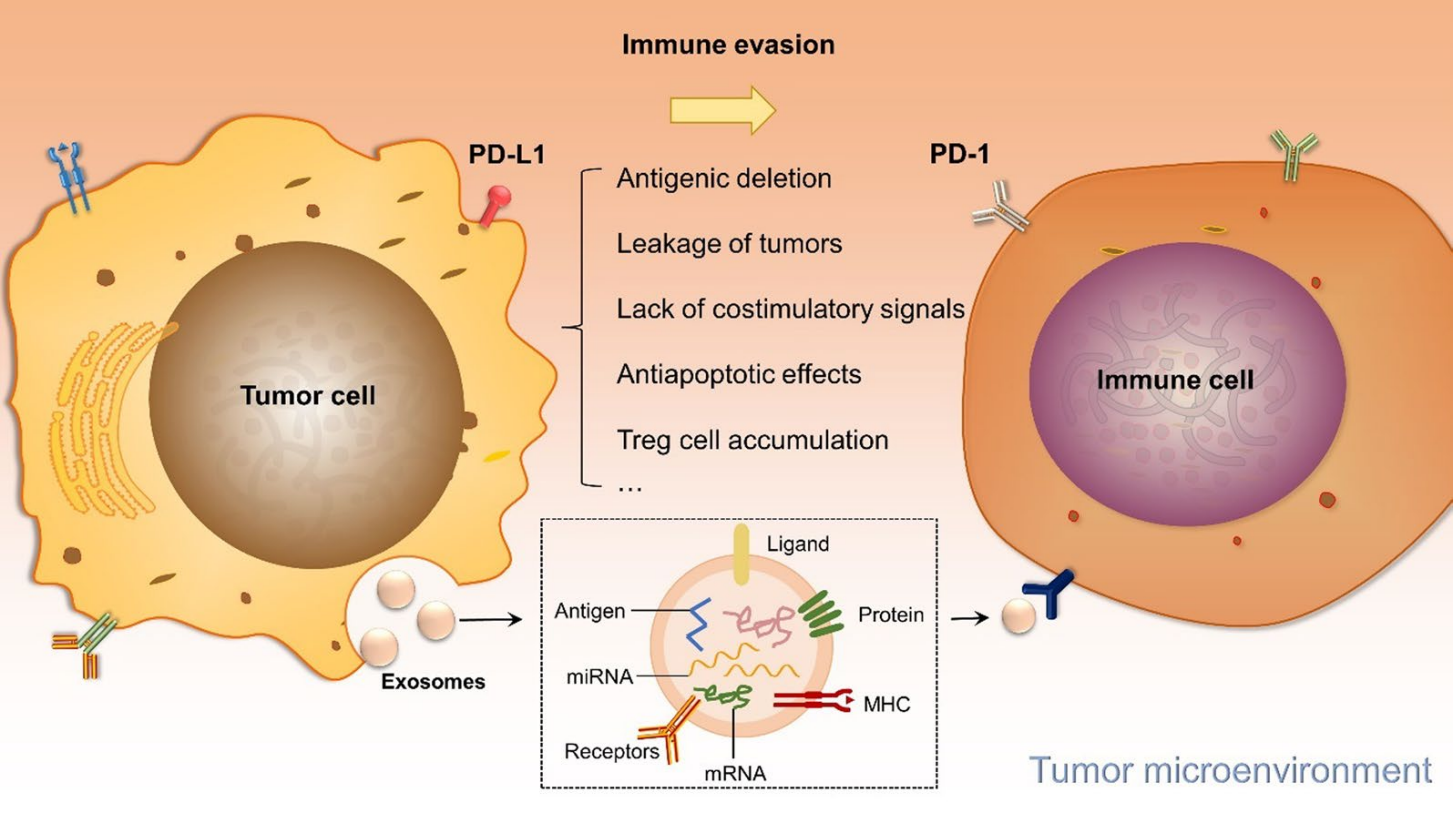
Proposed mechanism underlying tumor immune escape. Tumor immune escape is related to antigenic deletion, tumor cell leakage, lack of costimulatory signals on the tumor cell surface, and the antiapoptotic effects of tumor cells. Tumor cells bind to PD-1 on tumor-infiltrating lymphocytes via PD-L1 that mediates immune escape in the tumor microenvironment. Tumor cells also inhibit immune cells by releasing exosomes rich in surface PD-L1 to escape from immune system recognition

With developments in the fields of immunology, oncology, and molecular biology, our knowledge of tumor escape mechanisms is constantly expanding, yet many questions remain unanswered due to the diversity and complexity of tumors. For example, identification of additional molecular targets to reactivate immune cells and reverse the immunosuppressive state of the tumor microenvironment is an essential task in the field of antitumor immunity. As various tumor immune escape mechanisms exist as a part of a complex network, research and therapeutic development should avoid restricting analyses to certain antigen peptides, factors, and cell types. It is expected that a deeper understanding of tumor immune escape mechanisms will provide an extensive pool of information to enhance clinical immunotherapy development (Fig. 2).

## Hypoxia contributes to tumor immune escape

Rapid growth is a fundamental characteristic of tumors, and as a tumor increases in volume, its blood supply becomes insufficient, particularly in the core. Hypoxia is a characteristic trait of most solid tumors and an important feature of the solid tumor microenvironment that plays a pivotal role in tumor occurrence and development [16, 77]. In general, hypoxia in the local microenvironment promotes malignant behaviors, such as proliferation, infiltration, and metastasis. In-depth analysis of the hypoxic microenvironment will enhance our understanding of tumor growth and, more importantly, provide information regarding tumor diagnosis, treatment options, and prognosis [17, 78]. Accordingly, hypoxia has become a hot topic in the development of novel cancer treatment strategies [21].

The microenvironment of most solid tumors exists in a state of hypoxia, which is accompanied by activation of a series of related signaling pathways that enable tumor adaptation to the environment and enhance invasiveness and resistance to radiotherapy and chemotherapy, hindering treatment efficacy [25]. Therefore, the components of these signal transduction pathways have become popular topics in the study of tumor hypoxia, among which HIF1 has received the most attention. During hypoxia, HIF1A accumulates in the nucleus, activating hypoxia-regulated genes. Recognition of the importance of these discoveries has resulted in renewed interest in the effects of hypoxia on tumorigenesis [79].

Hypoxic regions often exist in solid tumors due to the rapid proliferation of tumor cells, insufficient blood supply, abnormal functions, and tumor blood vessel structure [80]. Moreover, as mentioned above, hypoxia plays a vital role in tumor immune escape mechanisms [81, 82]. Under hypoxic conditions, tumor cells release a large number of immunosuppressive molecules, including VEGF [83]. In addition to reducing the cytotoxicity of immune effectors, hypoxia alters the amounts of cell-surface immune checkpoint regulators and increases the intrinsic resistance of tumor cells to immune attack [84, 85]. Hypoxia-mediated angiogenesis is associated with immune tolerance [86]. Hypoxia induces expression of VEGF, which inhibits dendritic cell maturation [87, 88]; VEGF in turn enhances PD-L1 expression in dendritic cells, downregulating T cell function [89–91]. In addition, CD47 expression is related to HIF1A target VEGF. CD47 signaling regulates the VEGF immunosuppressive activity by interacting with VEGF receptor-2 [92, 93].Therefore, hypoxia directly suppresses the antitumor immune response, further enabling immune escape, as well as induces tumor cells to release immunosuppressive molecules [85, 94].

## HIFs: a crucial player in tumor immune escape

HIF1A is a critical transcriptional regulator that mediates the adaptive response of cells to the hypoxic microenvironment, promotes tumor angiogenesis, regulates metabolic reprogramming, accelerates the epithelial-mesenchymal transition (EMT), and increases radio-/chemotherapy resistance [88, 95]. Compared to severe hypoxia, mild to moderate hypoxia has a significantly greater effect on the efficacy of fractional radiotherapy in tumors, as severely hypoxic tumor cells are more likely to die. Hence, the level of HIF1A is related to the therapeutic effect of radiotherapy. Increased HIF1A expression is induced by reductions in nicotinamide adenine dinucleotide levels and downregulated sirtuin 1 expression under hypoxic conditions [96]. HIF1A is an important mediator of cells under hypoxia and is highly expressed in several tumors. To date, more than 100 HIF1 target genes have been identified, including those encoding proteins with functions closely related to cancer, such as angiogenic factors, glucose transport and glycolytic enzymes, and proteins involved in tumor invasion, metastasis, proliferation, and apoptosis [17].

Hypoxia contributes to tumor cell immune escape by inducing cytotoxic T lymphocyte (CTL) apoptosis through increased PD-L1 expression [10, 97], a process that is dependent on HIF1A signaling. For example, HIF1A-dependent increases in PD-L1 expression were observed when human cancer cells were exposed to hypoxia for 24 h [47]. Furthermore, in vivo studies have detected colocalization of HIF1A and PD-L1 in tumor cells [47]. By promoting apoptosis in CTLs and Jurkat (T-cell leukemia) cells, hypoxia-induced PD-L1 expression in tumor cells increases their resistance to CTL-mediated lysis [47].

Hypoxia also markedly increases PD-L1 expression in dendritic cells, macrophages, and tumor cells in an HIF1A expression-dependent manner [63]. Through chromatin immunoprecipitation assays to evaluate the *PD-L1* proximal promoter, HIF1A was found to bind directly to hypoxia response elements (HREs) to activate transcription [98]. Therefore, simultaneous blockade of PD-L1 and inhibition of HIF1A is a promising approach for cancer immunotherapy and should be thoroughly explored in the near future [7, 99].

In addition to PD-L1, VSIR, which is expressed on infiltrating hematopoietic cells (including MDSCs), is a negative immune checkpoint regulator in the B7 family of immunoregulatory ligands that suppresses T cell activation [100]. High VSIR expression in the hypoxic regions of a CT26 murine colon cancer model has been reported, in strong association with poor survival in patients with colon cancer [27]. Tumor hypoxia drives VSIR expression, which correlates significantly with HIF1A activity. Hypoxia-dependent *VSIR* mRNA and protein expression decrease in human peripheral mononuclear cells after HIF1A knockdown [27]. Thus, as another HIF1A target that contributes to tumor immune escape, HIF1A upregulates VSIR expression under hypoxia.

In one study, pancreatic tissues were collected from patients with pancreatic carcinoma or chronic pancreatitis, and isolated cells were cultured under hypoxic conditions [101]. HIF1A expression was significantly higher in pancreatic cancer cells than in control cells, including cells from patients with chronic pancreatitis or from those with healthy pancreatic tissue. In addition, HIF1A correlated negatively with major histocompatibility complex (*MIC*) class I chain-associated genes, which together with killer cell lectin-like receptor K1 (*KLRK1*) and hematopoietic cell signal transducer, activate immune surveillance by NK cells. That study revealed that MICs were shed from the pancreatic cancer cell membrane, providing a potential mechanism by which tumor cells evade KLRK1-mediated immune surveillance [101]. Hypoxia contributes to tumor cell MIC shedding through impairing nitric oxide (NO) signaling [102]. Although MIC shedding is increased by hypoxia in human prostate cancer cells, it is significantly inhibited after activation of NO signaling [101]. Hypoxia may also cause shedding of other NKG2D ligands, such as ULBPs. Overall, the role of HIF1A in this process warrants further investigation.

In hepatocellular carcinoma (HCC) cells, hypoxia is related to immunosuppressive Tregs recruitment through induction of C–C motif chemokine ligand 28 (CCL28) expression [103]. The supernatants of hypoxic HCC cells (SK-Hep-1, Hep3B, and HepG2 cells) cause significantly increased induction of Tregs migration compared with normoxic supernatants [103]. Moreover, CCL28 knockdown decreases hypoxia-induced Tregs recruitment, but CCL28 overexpression enhances recruitment; thus, CCL28 mediates Tregs recruitment under hypoxia. Furthermore, CCL28 transcript levels in HCC cell lines decrease after HIF1A knockdown. These data confirm that HIF1A upregulates CCL28 expression, resulting in recruitment of immunosuppressive CD4-, interleukin 2 receptor subunit alpha-, and forkhead box P3-positive Tregs, repressing T cell functions [104].

Hypoxia is a typical feature of prostate cancer, and a relationship between HIF1A-induced microRNA 224 (*miR224*) and natural cytotoxicity triggering receptor 1 (NCR1) has been uncovered in hypoxic prostate tumors [105]. *miR224* expression is significantly higher in prostate cancer tissues than in healthy prostate tissues; *miR224* is increased by HIF1A overexpression and suppressed by HIF1A knockdown, indicating that it is upregulated under hypoxia via HIF1A. In NK92 cells, the *NCR1* transcript level is significantly decreased after *miR224* transfection, whereas *NCR1* mRNA and protein levels increase after *miR224* inhibitor transfection [105]. HIF1A-induced *miR224* overexpression also attenuates NK cell cytotoxicity, dramatically decreasing the percentage of lysosomal-associated membrane protein 1-positive cells. Therefore, HIF1A-induced *miR224* overexpression inhibits NCR1 signaling, which helps in evasion of NK cell-mediated cytotoxicity.

A series of experiments were conducted to confirm that glutamine deficiency in ccRCC directly induces IL-23 secretion by tumor-infiltrating macrophages [28], and the role of HIF1A was explored to better understand the underlying signaling mechanism of glutamine deprivation-induced IL-23 secretion by macrophages. HIF1A is upregulated in murine macrophages with glutamine deficiency because the glutamine metabolite 2-oxoglutarate hydroxylates HIF1A, decreasing its transactivation activity [96]. HIF1A increases IL-23 expression in glutamine-deprived macrophages, and this effect is blocked by HIF1A inhibitor treatment [28]. Taken together, these results indicate that HIF1A expression in tumor-associated macrophages induces IL-23 secretion, which may suppress T cell immune functions through Tregs.

**Fig. 3:**
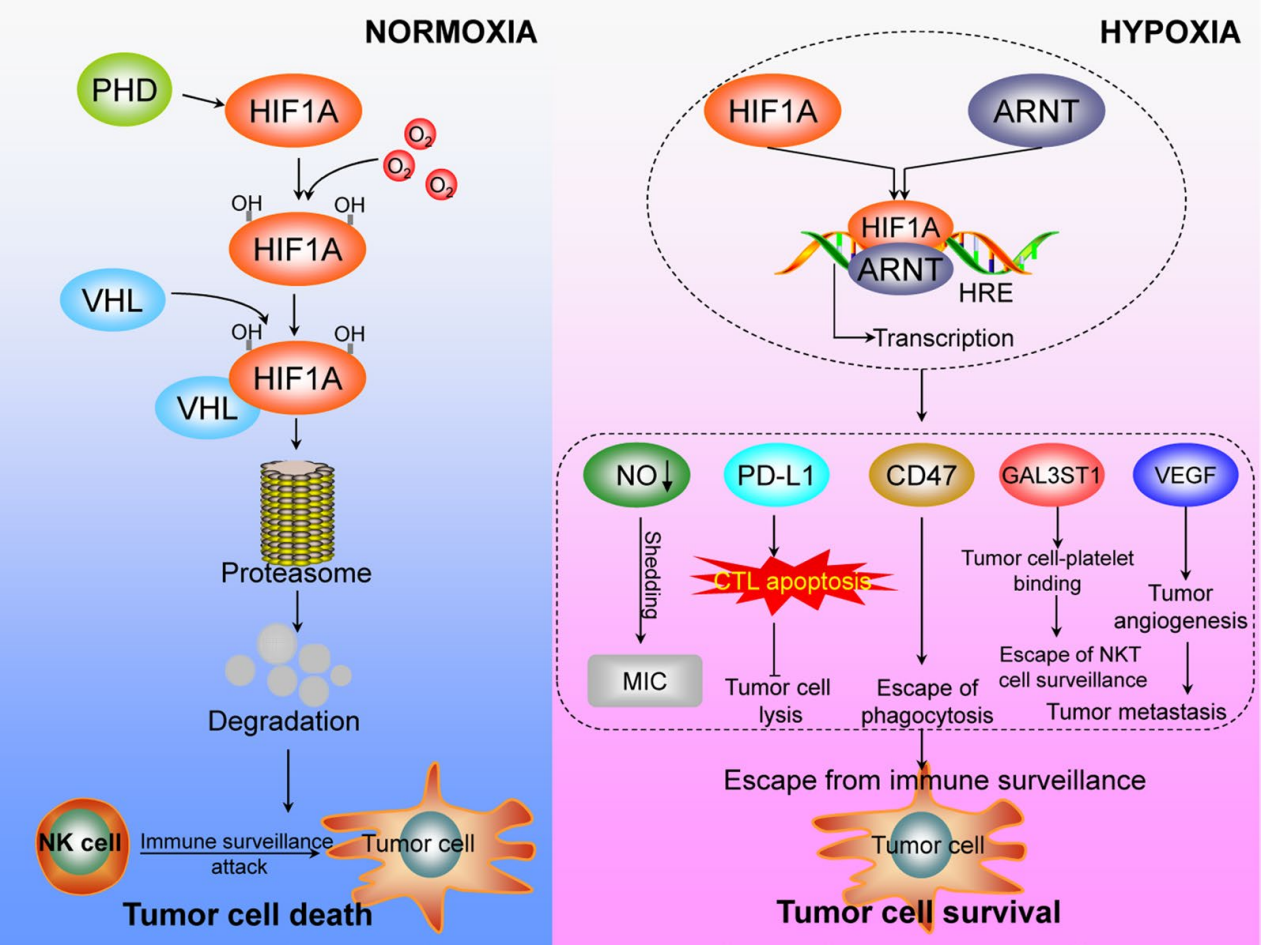
HIF1A regulates tumor immune escape under hypoxic conditions. Under normoxic conditions, HIF1A is hydrolyzed by prolyl-4-hydroxylase (PHD). Von Hippel-Lindau (VHL) then recognizes and binds to HIF1A, leading to rapid proteasomal degradation of HIF1A, rendering tumor cells susceptible to immune surveillance. Under hypoxic conditions, HIF1A is protected from degradation and translocates to the nucleus, where it dimerizes with hydrocarbon receptor nuclear translocator (ARNT) to form HIF1 and binds to target gene hypoxic response elements (HREs). HIF1 induces cytotoxic T lymphocyte (CTL) apoptosis by upregulating PD-L1 expression, which enhances tumor cell resistance to lysis. Moreover, HIF1A causes MIC shedding by impairing NO signaling, allowing escape from NK cell immune surveillance. The HIF1-GAL3ST1-sulfatide signaling axis promotes immune escape by increasing tumor cell-platelet binding. HIF1 also regulates CD47 expression to promote escape from phagocytosis and promotes tumor angiogenesis through upregulation of vascular endothelial growth factor (VEGF), which facilitates immune escape by enabling metastasis

The HIF1-galactose-3-O-sulfotransferase 1 (GAL3ST1)-sulfatide axis enhances immune escape in ccRCC by increasing tumor cell-platelet binding. Increased expression of GAL3ST1 in primary ccRCC correlates with decreased survival [106]. Moreover, *GAL3ST1* is an HIF1 target gene, and its expression is induced upon loss of von Hippel-Lindau (VHL) tumor suppressor, leading to accumulation of its enzymatic product sulfatide. Notably, platelets bind more efficiently to renal cancer cells with high GAL3ST1-sulfatide expression than to GAL3ST1-sulfatide-negative renal cancer cells, which protects ccRCC cells from NK cell-mediated cytotoxicity. Accordingly, *GAL3ST1* is an HIF1-responsive gene that contributes to ccRCC development by promoting tumor immune escape (Fig. 3).

HIF2A also has some functions in the mechanism of tumor immune escape [29]. Indeed, expression of HIF2A is associated with poor prognosis and low antitumor immune function in patients with renal cell carcinoma. In tumors with high HIF2A expression, the number of tumor-infiltrated CD8^+^ T cells is significantly reduced, showing a negative correlation [29]. In addition, tumor-infiltrating mast cells are related to immune tolerance promotion. Expression of characteristic genes (*GZMA*, *CCL5*, and *IL2RG*) and cytotoxicity of the adaptive immune system are significantly reduced in tumor-infiltrating mast cells [29]. HIF2A upregulates SCF expression in ccRCC, which promotes SCF recruitment of tumor-infiltrating mast cells. Moreover, upregulated expression of SCF protein increases secretion of IL-10 and TGF-β, resulting in the formation of an immunosuppressive tumor microenvironment and suppressed immune cell function [29]. Compared to HIF1A, data on the function of HIF2A in the tumor immune escape mechanism remain limited to date, and it has yet to be determined whether HIF2A may also be an upstream target to act on PD-L1/PD-1. In addition, we suspect that HIF2A may be similar to HIF1A and can regulate some downstream pathways, such as lncRNAs and CCL28, to achieve immune escape. Nevertheless, the mechanism of HIF2A in tumor immune escape remains to be fully elucidated.

The above studies clearly show that hypoxia activates multiple pathways and downstream target genes through HIF1A and HIF2A to enhance tumor immune escape and promote progression (Table 1). Blocking tumor immune escape by targeting HIFs is an active area of research.

**Table 1:** HIF signal pathways in tumor immune escape

Targets/effectors	Model	Mechanisms of tumor immune escape	Refs.
CD47	Human breast cell lines	HIF1 regulates CD47 expression to promote evasion of phagocytosis	[93]
VEGF	Human primary breast cells	HIF1A-induced VEGF correlates with PD-L1 expression	[91]
PD-L1	Human prostatic carcinoma cells Mouse B16-F10 melanoma	HIF1A upregulates PD-L1 expression in tumor cells causing T-cell apoptosis	[47]
VSIR	BALB/c mice CT26 colon carcinoma cell line	Hypoxia-induced VISTA, by HIF1A binding to the VISTA promoter, suppresses T-cell activity	[27]
MICs	The PANC-1 cell line Human pancreatic carcinoma cells	Hypoxia-induced MICs are shed from the tumors membrane to evade KLRK1-mediated immune surveillance	[101]
CCL28	Human haptic cell lines Hepatic cell lines Mouse hepatic cancer cells	HIF1A-dependently upregulates hypoxia-induced CCL28 to activate Tregs proliferation	[103]
IL-23	Human ccRCC tumor cells	Tumor cells induce tumor-infiltrating macrophages to secrete IL-23 by activating HIF1A, thereby inhibiting the killing capability of the cytotoxic lymphocytes	[28]
miR224	Human prostate cancer tissues	HIF1A upregulates miR-224 expression to inhibit the NK cells function by NCR1/NKp46 signaling	[105]
GAL3ST1	786-O, RCC4, HEK293A, HEK293T, NK-92 cells, primary kidney tissues	HIF1A and HIF2A upregulate the GAL3ST1 levels as VHL loss or hypoxia, further GAL3ST1 regulates sulfatide expression to escape the NK-mediated cytotoxicity	[106]
SCF	Human ccRCC tumor cells	HIF2A induces the SCF secretion thereby reducing immunosurveillance and impairing anti-tumor immunity	[29]

## LncRNAs in tumorigenesis and hypoxic tumor immune escape

Accumulating studies indicate that lncRNAs play pivotal roles in every stage of tumor progression and promote malignant behaviors, such as proliferation, migration, and invasion [107, 108]. In recent years, lncRNAs have also garnered widespread attention as novel factors in hypoxia [109]. The lncRNA *MIR31* host gene is overexpressed in oral squamous cell carcinoma and acts as an HIF1A coactivator, inducing HIF1 target genes and contributing to tumor development [110]. In osteosarcoma (OS) cells, HIF1A mediates overexpression of the lncRNA FOXD2 adjacent opposite strand RNA 1 (*FOXD2-AS1*) by binding to the promoter region. FOXD2-AS1 inhibits cyclin-dependent kinase inhibitor 1A (CDKN1A) expression by recruiting enhancer of zeste 2 polycomb repressive complex 2 subunit (EZH2) and is linked to a poor prognosis in patients with OS and promotes tumor progression [30]. FOXD2-AS1 overexpression promotes OS cell proliferation and is linked to a poor prognosis in OS [111]. We speculate that *FOXD2-AS1* overexpression is closely associated with the clinicopathological characteristics and prognosis of patients, suggesting it as a biomarker for tumor diagnosis and evaluating prognosis. However, research on HIF-induced *FOXD2-AS1* is still in its infancy. In addition to the hypoxic regulator, other signaling pathways regulate *FOXD2-AS1* [112, 113]. For example, treatment with IL-1β and TNF-α markedly induced overexpression of *FOXD2-AS1* and promote chondrocyte proliferation and inflammation, resulting in osteoarthritis [112]. Other signaling molecules, including *miR-4306*, regulate expression of *FOXD2-AS1* in colorectal cancer cells [114]. *FOXD2-AS1* is also expressed in other cancer cells, such as human glioma [113]. When the *FOXD2-AS1* gene was knocked out, the proliferation and migration of glioma cells were inhibited through *miR-1855p* regulation; *FOXD2-AS1* negatively regulates *miR-1855p*, thus promoting glioma tumorigenesis and progression [113]. The expression level of *FOXD2-AS1* in non-small-cell lung cancer is increased [115], and *FOXD2-AS1* overexpression promotes tumor cell proliferation and inhibits apoptosis in vitro and in vivo [115]. In hepatocellular carcinoma, *FOXD2-AS1* is highly expressed in tumor tissues, and its expression level is closely related to prognosis. Such high expression of *FOXD2-AS1* promotes tumor cell proliferation, invasion, and migration [116, 117]. The lncRNA urothelial carcinoma-associated 1 (*UCA1*) is involved in bladder tumor progression and identified as an oncogenic HIF1A target gene [31]. HIF1A binds to an HRE in the *UCA1* promoter to increase its expression. Under hypoxia, UCA1 inhibits apoptosis in bladder tumors cells and promotes their viability by modulating the BCL2-associated X, apoptosis regulator (BAX)/BCL2 apoptosis regulator (BCL2) ratio [31]. Hypoxia also induces production of exosomes containing *UCA1* in bladder tumors [118]. Compared with normoxic exosomes, secreted hypoxic 5637 (bladder carcinoma) cell-derived exosomes were found to have higher *UCA1* levels and to promote bladder tumor proliferation, migration, and invasion [118]. These results suggest that lncRNAs are vital mediators of HIF1-associated tumorigenesis.

**Fig. 4:**
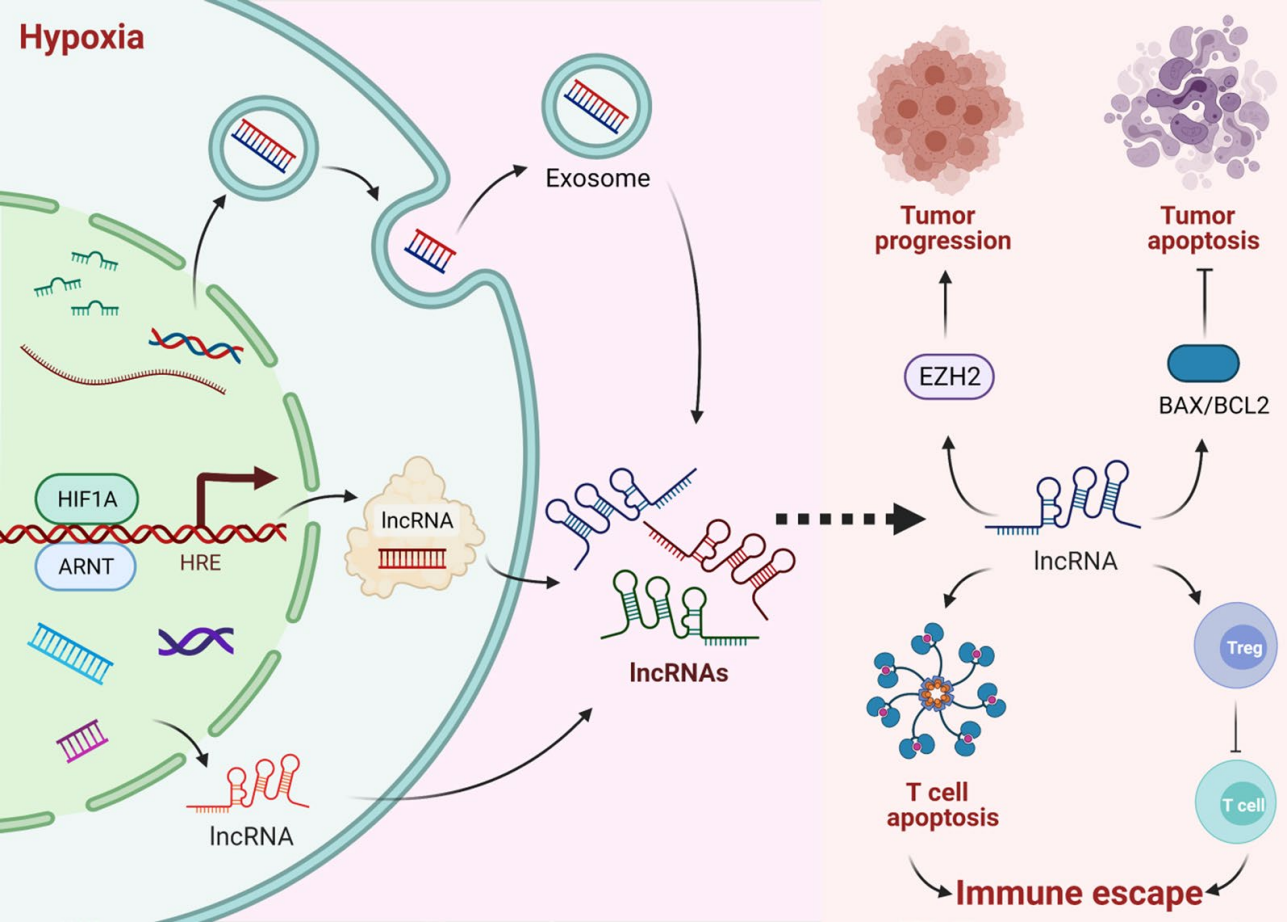
Effects of lncRNAs on hypoxic tumor immune escape. HIF1A induces lncRNA overexpression, inhibiting cyclin-dependent kinase inhibitor 1A (CDKN1A) expression by recruiting enhancer of zeste 2 polycomb repressive complex 2 subunit (EZH2) and causing tumor progression. Under hypoxia, lncRNAs inhibit tumor cell apoptosis by regulating the BAX/BCL2 ratio. Hypoxia induces production of lncRNA-containing exosomes in tumor cells and promotes proliferation, migration, and invasion. Notably, lncRNAs also contribute to immune escape by increasing Tregs levels, inhibiting CTLs, and modulating the sensitivity of tumor-infiltrating T cells to apoptosis

Notably, lncRNAs are also involved in immune escape, with effects on Tregs, CTLs, and the PD-L1/PD-1 immune checkpoint [119], and may represent novel antitumor targets. EGFR antisense RNA 1 (*EGFR-AS1*) expression correlates with Tregs levels and inhibits CTLs in HCC, contributing to tumor immune escape. *EGFR-AS1* overexpression is significantly increased Tregs, a situation that is reversed by *EGFR-AS1* knockdown [119]. In breast cancer, an NF-κB-interacting lncRNA modulates T cell sensitivity to apoptosis, shifting the balance between CTLs and immunosuppressive Tregs in the tumor microenvironment and resulting in tumor immune escape [120]. Small nucleolar RNA host gene 14 (*SNHG14*), which interacts with *miR-5590-3p*, is upregulated in diffuse large B-cell lymphoma (DLBCL) cells, where it triggers CD8^+^ T cell apoptosis and enhances DLBCL growth by activating the PD-L1/PD-1 immune checkpoint [121]. Nonetheless, it remains unclear whether HIF1A can regulate these lncRNAs in immune escape and therefore may be a promising target for immunotherapy, though we hypothesize that the lncRNAs induced by HIFs are involved in immune escape. The proposed mechanisms by which lncRNAs affect hypoxic tumor immune escape are illustrated in Fig. 4.

## HIFs regulate innate immunity

Innate immunity influences cancer progression [122, 123], and increasing lines of evidence show that HIFs regulate innate immunity [124, 125]. In the innate immune system, the HIF response is the same as its role in hypoxia [126] and is associated with various inflammatory diseases and immunosuppression [78]. In a diethylnitrosamine-induced mouse liver cancer model, loss of HIF2A in myeloid cells inhibits invasion of liver cancer by tumor-associated macrophages [127]. HIF2A drives expression of receptors in myeloid cells, such as CXCR4, M-CSFR, and fibronectin 1, enabling them to migrate to and infiltrate sites of inflammation [127]. Furthermore, HIF1A-induced miR-210 expression in tumor-related MDSCs is higher than that in splenic MDSCs. In tumor-associated MDSCs, miR210 increases ARG1 and decreases IL-16 and CXCL12 to suppress the immune function of T cells [128]. HIF1A may cause anti-inflammatory polarization of neutrophils. HIF1 activates the NF-κB pathway in liver cancer cells and promotes CXCL5 secretion, further attracting tumor-associated neutrophils to liver cancer [129, 130]. Liver cancer cells activate the PI3K/AKT and p38/MAPK pathways in tumor-associated neutrophils via HIF1 and induce expression of CCL2 and CCL17 [129]. In addition to attracting immunosuppressive cells, tumor-associated neutrophils promote tumor angiogenesis through HIF1A and correlate significantly with the number of M2 macrophages and Tregs and poor prognosis, suggesting that tumor-associated neutrophils have an immunosuppressive effect in liver cancer [129].

## Functional regulation of HIFs under normoxia

HIFs also play a role in transcriptional regulation in nonhypoxic environments [131]. Moreover, HIF1A stabilization in immune cells occurs in an oxygen-dependent or oxygen-independent manner [122, 132]. Under normal physiological oxygen conditions, the stability and transcriptional activity of the HIF1A protein is significantly increased by a series of related factors, such as platelet-derived growth factor and epidermal growth factor. Regulation of its nonoxygen-dependent activity mainly occurs through two signal transduction pathways: the Ras/MAPK and PI3K/AKT kinase cascades [133]. Under normoxic conditions, innate immune B cells stimulated by lipopolysaccharide induce expression of HIF1 mRNA but not that of HIF2 mRNA. HIF1A and STAT3 cooperatively regulate IL-10 transcription via HRE I and HRE II regions to exacerbate autoimmune diseases and cancer metastasis [134]. In normoxia, HIFs are hydroxylated by prolyl hydroxylases and bind to the oncosuppressor protein VHL [135]. HIF2A mRNA and protein levels in VHL-deficient ccRCC lines are elevated, which suggests that HIF2A is regulated by the transcription level and protein stability of *VHL* gene products [136]. In general, HIFs significantly influence cancer invasiveness [137]. For example, HIF1A interacts with GATA binding protein 3 to contribute to enhanced tumor cell invasiveness in head and neck squamous cell carcinoma [138], and expression of HIF2A and tumor thymidine phosphorylase under the action of an oxygen-dependent pathway shows an inverse correlation, eventually resulting in tumor angiogenesis and invasion [139].

## HIFs in cancer invasiveness and metastasis

Tumor invasiveness and metastasis, the main risk factors affecting the prognosis of patients [34], is a continuous event of uncontrolled cell proliferation, angiogenesis, separation, movement, deposition in microvessels, extravasation from blood vessels and proliferation at secondary sites [34]. In neuroblastoma cells, HIF1A regulates the sonic hedgehog signaling pathway to promote the invasive abilities of cancer cells [38]; in human osteosarcoma tissue, expression of differentiated embryonic chondrocyte gene 2 (DEC2) and HIF1A is closely related to poor prognosis [37]. HIF1A upregulates DEC2 at the transcriptional level under hypoxic conditions, which in turn promotes HIF1A activation. This uncontrolled HIF1A activation, which promotes HIF1A expression with DEC2, ultimately contributes to the transcriptional reprogramming, metabolic reprogramming, angiogenesis, and invasiveness that occurs in osteosarcoma [37]. Liver cancer cells secrete soluble stem cell factors to promote angiogenesis of vascular endothelial cells [39], and SCF overexpression in liver cancer cells is regulated by HIF2A-dependent mechanisms. Knockout of HIF2A significantly reduces expression of SCF; HIF2A directly induces transcription of the *SCF* gene via the hypoxia response element in the *SCF* promoter to upregulate its expression, thereby promoting angiogenesis and metastasis in hepatocellular carcinoma [39].

Because it is a complex and dynamic process, metastasis is a major challenge in the clinical treatment of tumors [35, 140]. Zinc finger MYND-type containing 8 (*ZMYND8*) is a direct target gene of HIF1A and HIF2A [141]. In breast cancer cells, ZMYND8 interacts with HIF1A and HIF2A by binding to the HREs H3K14ac and H4K16ac; subsequently, the ZMYND8/HIF axis increases breast tumor angiogenesis and decreases cancer cell death to promote metastasis [141]. In VHL-deficient and hypoxic ccRCC tumor cells, protein kinase growth arrest-specific 6 (GAS6)/AXL is activated by HIF1A and HIF2A. SRC proto-oncogene nonreceptor tyrosine kinase is a direct target of GAS6/AXL signaling, and once activated, it induces the MET proto-oncogene, thereby regulating EMT and ccRCC tumor metastasis [142]. In addition, the HIF1A protein is overexpressed in hypoxic tumors; the cancer stem cell marker CD24 is also overexpressed in many tumors, confirming the correlation whereby HIF1A leads to CD24 overexpression in hypoxic tumors [143]. Overexpression of HIF1A promotes production of CD24, which leads to tumor growth and metastasis. CD24 also promotes aggressive growth and metastasis characteristics in tumors. In addition to making tumors more aggressive, it has been verified that CD24 renders tumors resistant to chemotherapy, resulting in tumor recurrence and deterioration after chemotherapy [143].

**Fig. 5:**
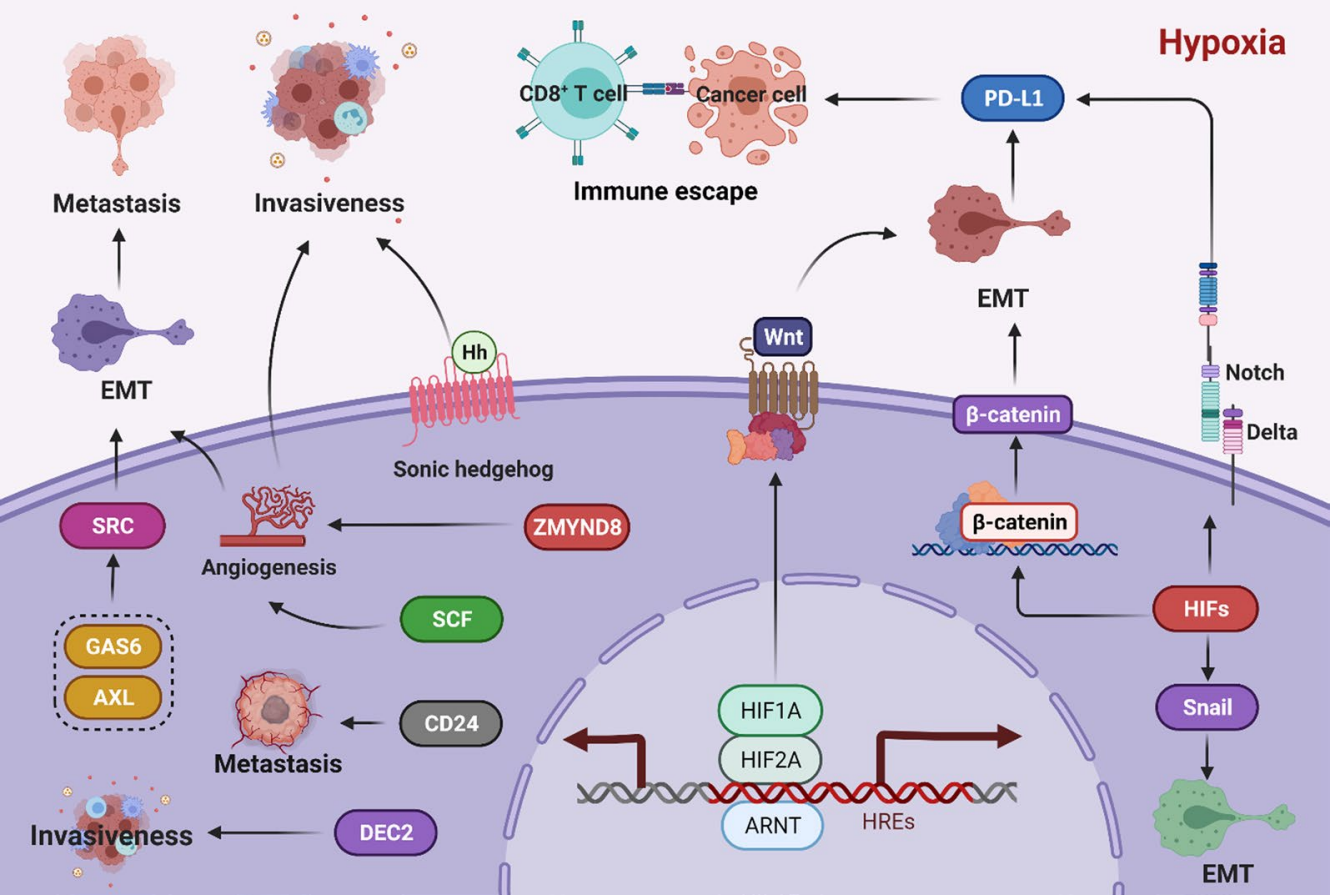
The role of HIFs in cancer invasiveness and metastasis. HIF1A regulates the sonic hedgehog signaling pathway to promote the invasive abilities of cancer cells. Under hypoxic conditions, HIF1A upregulates differentiated embryonic chondrocyte gene 2 (DEC2) at the transcriptional level, which in turn promotes HIF1A activation, ultimately contributing to cancer cell metabolic reprogramming, angiogenesis, and invasiveness. HIF2A directly induces transcription of the *stem cell factor* (*SCF*) gene via the hypoxia response element in the *SCF* promoter and upregulates SCF expression, thereby promoting angiogenesis and metastasis. Zinc finger MYND-type containing 8 (ZMYND8) is regarded as a direct target gene of HIF1A and HIF2A. The ZMYND8/HIF axis increases breast tumor angiogenesis and decreases cancer cell death to promote cancer metastasis. Protein kinase growth arrest-specific 6 (GAS6)/AXL is activated by HIF1A and HIF2A. The SRC proto-oncogene nonreceptor tyrosine kinase is a direct target of GAS6/AXL signaling; it activates MET proto-oncogenes, thereby regulating the epithelial-mesenchymal transition (EMT) and tumor metastasis. Overexpression of HIF1A promotes production of CD24, which leads to tumor growth and metastasis, and HIFs regulate EMT to promote cancer metastasis. Hypoxia-induced HIFs promote EMT by enhancing snail, β-catenin, Wnt, and Notch signaling, thereby inducing cancer cell survival, metastasis, and immune escape. EMT promotes expression of PD-L1, facilitating tumor cells escape and recognition and attack by immune cells

HIFs regulate EMT to promote cancer metastasis [144]. EMT refers to the biological process in which epithelial cells transform into cells with a mesenchymal phenotype through specific processes [145]. Hypoxia-induced HIF1A promotes EMT by enhancing the snail, β-catenin and Notch signaling pathways, leading to cancer cell survival, cancer metastasis and resistance to immune attack [144, 146]. HIF2A also regulates EMT by activating the Wnt and Notch pathways and promotes a stem cell phenotype in breast cancer cells as well as chemotherapy resistance [147]. In non-small-cell lung carcinoma, cytokine TNF-α and TGF-β1-coinduced EMT promotes expression of PD-L1 and is involved NF-κB signaling, suggesting a link between EMT and immune escape [148]. Hence, EMT mediated by the cytokines TNF-α and TGF-β1 is synergistic, rather than acting alone, to increase expression of PD-L1. Other signaling pathways that influence expression of PD-L1 may exist, and we speculate that HIFs first regulate EMT and then promote expression of PD-L1, ultimately leading to immune escape (Fig. 5).

HIFs also play crucial roles in cancer stem cells [78, 125–128], a small number of cells with self-renewal ability, multidifferentiation potential, and tumorigenic properties that are present in tumor tissue when a tumor is transplanted into an animal host [149]. HIF1A transforms normal stem cells into cancer stem cells by altering the niche components and functions of stem cells, leading to an increase in drug-resistant cancer stem cells in solid tumors [150], and HIF1A- and HIF2A-dependent AlkB homolog 5 expression induces the breast cancer stem cell phenotype [151]. A transcriptional regulator in early embryos and undifferentiated embryonic stem cells, octamer binding transcription factor 4 (Oct-4) regulates expression and transcription activities by HIF2A [152]. Under hypoxic conditions, cancer stem cells activate the Oct-4 pathway through high HIF2A expression, thereby maintaining their characteristics [152, 153]. Importantly, under hypoxic conditions, HIF2A silencing inhibits the phenotype of glioblastoma stem cells and promotes that of differentiated cells [154]. HIF2A also induces tumor stem cells and is complementary to existing DNA alkylation therapy in inhibiting the activity of glioblastoma stem cells [154].

## HIFs regulate tumor chemoresistance

ATP binding cassette subfamily B member 1 (ABCB1) is an efflux drug transport protein located in the cell membrane that is responsible for excreting drugs from tumor cells, resulting in multidrug resistance [155]. The transmembrane drug efflux transporter P-glycoprotein in ABCB1 is closely related to multidrug resistance and contributes to efflux of antitumor drugs under hypoxic conditions [155]. Interestingly, *ABCB1* is an HIF1A target gene, and HIF1A participates in the hypoxia-mediated multidrug resistance of tumor cells by inducing ABCB1 expression, reducing chemotherapeutic drug accumulation in tumor cells and inhibiting chemotherapy-induced apoptosis [156]. HIF1A and TGF-β2 jointly activate the tumor resistance gene *GLI2*, enhancing intrinsic tumor resistance to chemotherapeutic drugs [157]. Both ABCB1 and HIF1A are upregulated under hypoxic conditions, suggesting that HIF1A is related to tumor radiotherapy and chemotherapy resistance mechanisms [158].

In gastric cancer cells, HIF1A induces multidrug resistance via *miR-27a* [159]. Expression of HIF1A is enhanced in gastric cancer cells, and that of HIF1A is highest in drug-resistant gastric cancer cells. These results suggest that HIF1A is involved in the development of multidrug resistance in gastric cancer cells [159]. HIF1A positively regulates olfactomedin 4, leading to hypoxia-induced invasion, EMT, and chemotherapy resistance in non-small-cell lung cancer cells [160]. Moreover, HIF1A and HIF2A are critical for cisplatin resistance. In lung cancer cells, HIF1A and HIF2A induce cisplatin resistance by enhancing autophagy induction under hypoxic conditions [161]. HIF2A mediates oxaliplatin resistance in colon cancer cells. Oxaliplatin also increases HIF2A accumulation, which leads to enhanced cancer cell growth [162]. In hypoxic liver cancer cells, sorafenib induces upregulation of HIF2A expression, and HIF2A participates in drug resistance by activating the TGF-α/EGFR pathway [163]. Therefore, HIFs may induce drug resistance.

## Targeting HIFs in cancer therapy

In view of the important role of HIFs in tumor growth, metastasis, invasion, and immune escape, treatment methods targeting HIFs have attracted much attention [164, 165]. Drugs that inhibit HIF activity mainly include signal transduction pathway inhibitors and small molecule inhibitors [166, 167]. 17-allyl-amino-geldanamycin inhibits the activity of HSP90, which interacts with HIF1A to induce its degradation via a VHL-independent pathway [40, 168, 169]. It is worth noting that in esophageal squamous cell carcinoma, HIF1A, COX-2, and PD-L1 show high expression levels and are associated with poor prognosis. The HIF1A inhibitor PX-478 suppresses tumor growth, induces cell cycle arrest in G2 phase, promotes cancer cell apoptosis, and reduce expression of COX-2 and PD-L1 [170]. Moreover, the redox regulator thioredoxin-1 promotes HIF1A expression. Therefore, thioredoxin inhibitors suppress the growth of xenograft tumors by reducing expression of HIF1A [41, 171]. Although research on specific inhibitors of HIF2A is limited compared to that on HIF1A, the HIF2A inhibitor PT2385 not only suppresses expression of HIF2A in renal cell carcinoma metastases but also reduces it in normal tissues. This inhibitor induces dissociation of HIF2A heterodimers to inhibit HIF2A target genes, involving tumor-suppressor genes in renal cell carcinoma [172]. In addition, the small molecule PT2399 directly inhibits HIF2A and causes tumor regression in a targeted manner in preclinical models of primary and metastatic pVHL-deficient renal cell carcinoma [173].

Some inhibitors achieve therapeutic effects by inhibiting expression of HIF messenger ribonucleic acid or protein, dimerization, and transcriptional activity [95, 164]. Aminoflavone, aromatic hydrocarbon receptor ligands, effectively inhibit expression of HIF1A mRNA in breast cancer cells and almost completely block accumulation of the HIF1A protein as well as transcription of downstream target genes in an aromatic hydrocarbon receptor-independent manner [174]. However, its clinical application has not been confirmed [164]. Acriflavine binds directly to HIF1A and HIF2A domains and is an effective inhibitor of dimerization; treatment with acriflavine effectively reduces tumor growth and angiogenesis in prostate and hepatocyte xenograft models [175]. Chetomin destroys the zinc binding site in the CH1 domain of p300, hindering HIF-p300 interaction; it prevents binding of HIF1A and HIF2A to p300, with an antitumor effect in vivo [176].

With the maturity of DNA recombination technology, gene therapy is a new strategy for tumor treatment because it corrects mutations or defective genes by introducing genes into target cells [167]. *miR-107* regulated by the *p53* gene inhibits expression of HIF1A and inhibits hypoxic signaling [177, 178]. *HIF1A* siRNA was transfected into HepG2 cells under hypoxic conditions, downregulating HIF1A and its target gene *VEGF* at the mRNA and protein levels, with an antiangiogenic effect on liver cancer [179]. As suppression of tumor growth by inhibiting transcriptional activation of HIF target genes is one of the most effective anticancer strategies, HIFs might be used as a molecular target for the development of antitumor drugs. However, further work is needed with regard to the construction and modification of gene therapy vectors, especially how to make retroviruses safer and more effective. The major HIF targeted agents in cancer therapy are summarized in Table 2.

**Table 2:** HIF targeted agents in cancer therapy

Agents	Model	Regulatory mechanisms	Refs.
17-allyl-amino-geldanamycin	Human glioma cell lines	Geldanamycin decreases cancer cell migration via HIF1A degradation	[169]
PX-478	Esophageal squamous cancer cells	HIF1A inhibitor PX-478 promotes apoptosis and inhibits G2/M transition contributing to tumor cell proliferation	[170]
PX-12	HT-29 colon carcinoma cell line	Thioredoxin-1 inhibitor decreases the tumor vascular permeability by downregulating HIF1A protein levels and VEGF expression	[41]
PT2385	Human ccRCC tumor cells	HIF2A inhibitor PT2385 hinders cancer metastases by dissociating HIF2A complexes	[172]
PT2399	786-O cells Human ccRCC tumor cells	HIF2A small molecule inhibitor PT2399 causes tumor regression	[173]
AFP464	Human cancer cells lines	Aminoflavone inhibits HIF1 and HIF2 transcriptional protein accumulation for anti-cancer activity	[174]
Acriflavine	HEK293 cells Human P493 cells	Acriflavine inhibits the HIF1 dimerization and has inhibitory effects on tumors growth	[175]
Chetomin	HCT116 cells	Epidithiodiketopiperazines could inhibit the HIF1A-p300 binding via zinc ion ejection	[176]

## Conclusion

Although the crucial role of hypoxia in cancer has been demonstrated, the precise effects of hypoxia and HIFs on tumor immune escape are not fully understood. Tumor immune escape is a multifaceted process that involves both inhibition of immune effectors and the intrinsic resistance of tumor cells to immune effectors. By modulating tumor cell-intrinsic characteristics and the tumor matrix composition, hypoxia participates in many aspects of tumor immune escape. As such, the main regulators of the hypoxia response, HIF1A and HIF2A, are promising therapeutic targets.

Overall, clarifying how tumors achieve immune escape is of great significance for identifying clinically relevant drug targets. Although knowledge regarding tumor immune escape mechanisms is growing, most current therapeutic agents target only one tumor immune escape mechanism. In contrast, tumor occurrence and development are complex, dynamic, and continuous processes resulting from various combinations of immune escape mechanisms.

The metastasis, invasion and drug resistance of malignant tumors are closely related to HIFs, which regulate angiogenesis, supply nutrients to the hypoxic area of malignant tumors, and promote tumor proliferation. Moreover, HIFs regulate oncogenes and tumor-suppressor genes under oxygen-dependent and nonoxygen-dependent conditions, such that they are overexpressed in malignant tumors and affect innate immunity by regulating target genes (*DEC2*, *ZMYND8*, and *CD24*), promoting tumor invasion, metastasis and immune escape. Overexpression of HIF1A and HIF2A also accelerates excretion of chemotherapeutic drugs from tumor cells and induce abnormal DNA damage repair in cells, thereby reducing the sensitivity of cells to chemotherapeutic drugs based on DNA damage, resulting in tumor multidrug resistance. However, due to the complexity of the tumor cell microenvironment, current research on the mechanism of HIF-induced tumor resistance remains limited.

Although several hypoxia-targeted drugs have achieved good results in clinical trials, many challenges remain to be addressed. As with all antitumor drugs, hypoxia-targeted drugs will inevitably result in acquired resistance, allowing reactivation of HIF signaling. In addition, hypoxia-targeted therapies have variable effects on different patients and tumor types. The design of new inhibitors targeting HIFs and their various signal transduction pathways has been an area of interest in recent years, and because all tumor cells are hypoxic and hypoxic tumor cells are highly tolerant to radiotherapy, combined use of HIF inhibitors and radiotherapy can improve the efficacy of radiotherapy.

In this work, we explored the mechanisms underlying how hypoxia, HIFs, and lncRNAs promote tumor immune escape. Under hypoxic conditions, HIF1A induces activation of target genes (*PD-L1*, *CCL28*, and *GAL3ST1*), resulting in immune cell apoptosis, and drives expression of the negative immune checkpoint regulator VSIR and MIC genes to evade immune surveillance and ultimately promote tumor immune escape. HIF2A plays an important role in the tumor immune escape mechanism and promotes SCF secretion and recruits mast cells in ccRCC patients. HIF activation also exhibits anti- and proinflammatory effects regulating the activity of immune cells, and HIF1A can induce nonalcoholic steatohepatitis and further cause autophagy damage to macrophages by mediating NF-κB activation and MCP-1 production. HIFs have a key role in innate immunity, especially in regulating CXCR4, M-CSFR and *miR-210*. In addition, lncRNAs can serve as HIF1A downstream signaling mediators.

We discussed the involvement of lncRNAs in immune escape mediated through suppression of T cell immune functions, activation of the PD-L1/PD-1 immune checkpoint and recruitment of immunosuppressive Tregs. However, the detailed molecular mechanisms by which hypoxia-related lncRNAs promote immune escape remain largely unknown. We believe that understanding the intertwined roles of hypoxia, HIFs, and lncRNAs in tumor immune escape will help to elucidate the highly complex immune response mechanism and provide important clues to further enhance existing tumor immunotherapies.

## Data Availability

Not applicable, all information in this review can be found in the reference list.

## Abbreviations

ABCB1: ATP-binding cassette subfamily B member 1
CCL28: C–C motif chemokine ligand 28
ccRCC: Clear cell renal cell carcinoma
CDKN1A: Cyclin-dependent kinase inhibitor 1A
CTL: Cytotoxic T lymphocyte
DEC2: Differentiated embryonic chondrocyte gene 2
DLBCL: Diffuse large B-cell lymphoma
EMT: Epithelial-mesenchymal transition
EZH2: Zeste 2 polycomb repressive complex 2 subunit
HCC: Hepatocellular carcinoma
HIF1A and HIF2A: Hypoxia-inducible factors (HIFs) 1 and 2 alpha
HREs: Hypoxic response elements
IL-10: Interleukin-10
KLRK1: Killer cell lectin-like receptor K1
LncRNAs: Long noncoding RNAs
MIC: Major histocompatibility complex class I chain-associated
NCR1: Natural cytotoxicity triggering receptor 1
NO: Nitric oxide
Oct-4: Octamer binding transcription factor 4
OS: Osteosarcoma
PD-1: Programmed cell death protein 1
PD-L1: Programmed cell death 1 ligand 1
SCF: Stem cell factor
SNHG14: Small nucleolar RNA host gene 14
TGF-β: Transforming growth factor-β
Tregs: Regulatory T cells
UCA1: Urothelial carcinoma-associated 1
VEGF: Vascular endothelial growth factor
VHL: Von Hippel-Lindau
VSIR: V-set immunoregulatory receptor
ZMYND8: Zinc finger MYND-type containing 8
